# Origami-Type Flexible Thermoelectric Generator Fabricated by Self-Folding

**DOI:** 10.3390/mi14010218

**Published:** 2023-01-15

**Authors:** Yusuke Sato, Shingo Terashima, Eiji Iwase

**Affiliations:** School of Fundamental Science and Engineering, Waseda University, 3-4-1 Okubo, Shinjuku-ku, Tokyo 169-8555, Japan

**Keywords:** flexible device, origami, TEG, self-folding, linkage mechanism

## Abstract

The flexibility of thermoelectric generators (TEGs) is important for low-contact thermal resistance to curved heat sources. However, approaches that depend on soft materials, which are used in most existing studies, have the problem of low performance in terms of the substrate’s thermal conductivity and the thermoelectric conversion efficiency of the thermoelectric (TE) elements. In this study, we propose a method to fabricate “Origami-TEG”, a TEG with an origami structure that enables both flexibility and the usage of high-performance rigid materials by self-folding. By applying the principle of the linkage mechanism to self-folding, we realized a fabrication process in which the TE element-mounting process and the active-material-addition process were separated in time. The fabricated origami-TEG showed similar internal resistance and maximum output power when attached to heat sources with flat and curved surfaces. Furthermore, it exhibited high-performance stability against both stretching and bending deformations.

## 1. Introduction

Thermoelectric generators (TEGs), which can use waste heat from the environment, are useful as independent power sources for wireless sensors for the Internet of Things (IoT) [1,2,3,4,5,6,7,8,9,10,11,12,13]. Recently, flexible TEGs have attracted attention because they can attach to various shapes of heat sources, such as hot pipes in factories and human skin [3,4,5,6,7,8,9,10,11,12,13]. In general, there are two ways to provide flexibility to electronic devices: material- and structure-based approaches. Material-based approaches include using soft polymers, such as polydimethylsiloxane (PDMS) or hydrogels, as the substrates or fabricating elements from soft organic materials [7,8,9,10,11,12,13,14]. Although these methods are effective in terms of biocompatibility and stretchability, flexible materials have the critical problems of low thermal conductivity and TE performance. Therefore, their application in TEGs should be handled with caution. In contrast, structure-based approaches include reducing the device’s thickness, weaving in fibrous materials, and creating a folded structure on the substrate [15,16,17,18]. In particular, providing origami structures such as bellows folds on the substrate is a promising method for realizing flexible TEGs because it enables both the flexibility of the entire device, owing to the substrate structure, and the usage of rigid and high-performance inorganic TE elements. However, the problem is the yield reduction due to manual manufacturing when the origami structure becomes finer and larger in area. Therefore, a simple manufacturing method that does not depend on the manual folding of a TEG with an origami structure (origami-TEG) is necessary.

In this study, we fabricated an origami structure of a substrate by self-folding and realized an origami-type flexible TEG that yields the mounting of rigid TE elements with high thermal-conversion efficiency. Self-folding is a technique of spontaneously folding structures using the deformation of active materials, such as heat-shrinkable films, in response to external stimuli, such as heat, and it effectively fabricates origami structures that are difficult to fold manually [19,20,21]. Generally, it is difficult to form a bellows fold using the conventional self-folding method for devices such as TEGs, which have TE elements sandwiched between the top and bottom substrates, because the active material cannot be pre-assembled into the substrate because of the negative effects on the active material caused by thermal stimulation from the mounting of the element. Therefore, we propose a method to fold origami-TEG into the target origami structure using the theory of linkage mechanism to the self-folding process, even if the fabrication procedure is to attach the shrink layer after the element-mounting process. By modeling the structure as a linkage mechanism, calculating the theoretical driving force required to achieve the target shape, and considering how to control the driving force generated by the hinge, the device self-folds into the target origami shape. To evaluate the origami-TEG, we measured the basic characteristics when the temperature of the heat source was changed and the output power and resistance change rate when the origami-TEG was deformed by stretching and bending, confirming the realization of a flexible TEG with high resistance to deformation. 

## 2. Theory and Design

**Figure 1:**
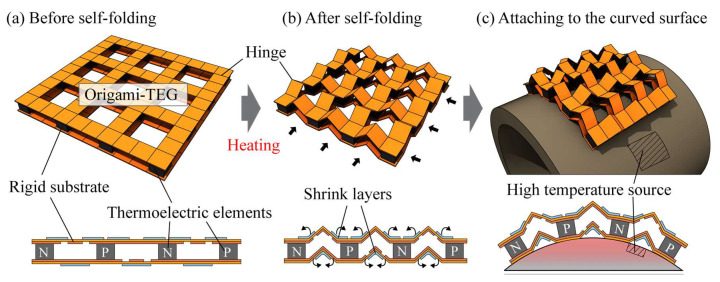
Schematic of origami-type flexible thermoelectric generator (origami-TEG). (**a**) Origami-TEG before self-folding. (**b**) Origami-TEG after self-folding. The shrink layer generates a driving force to fold up by heating and forms an origami structure spontaneously. (**c**) Origami-TEG attached to a curved heat source. The bellows fold structure of the substrate arranged in a grid pattern gives the TEG high flexibility.

Figure 1 is a schematic of the origami-TEG fabricated in this study. First, as shown in Figure 1a, the TE element was mounted between the top and bottom substrates with a slit in the folded area to reduce bending rigidity. The shrink layer should be attached from the outside after all stimulating fabrication processes, such as device mounting, to prevent unexpected folding caused by the heat-shrinkable film used for self-folding. The design should enable the attachment of the shrink layer using a linkage mechanism to ensure all hinges of the origami-TEG can be folded up to the target geometry. Heating the entire device causes the shrinking layer to generate a driving force that folds the hinge up, deforming it into the origami structure shown in Figure 1b. The hinge part of the bellows structure can be deformed to various angles even after self-folding. Therefore, as shown in Figure 1c, the origami-TEG, which consists of a grid-like bellows structure, has high stretchability and bendability even if the mounted TE element is rigid, and can follow a curved high-temperature source with any shape with low-contact heat resistance.

**Figure 2:**
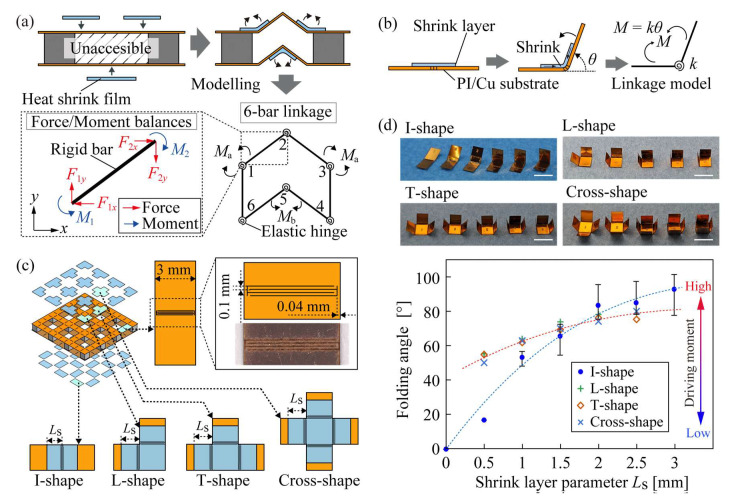
Design methods for self-folding using linkage mechanisms. (**a**) Modeling of a pair of origami-TEG. Force and moment analysis considering the linkage mechanism. (**b**) Modeling of hinges. (**c**) Design of hinge specimens and definition of shrink layer parameter *L*_s_. (**d**) Relationship between shrink layer parameter *L*_s_ and folding angle *θ* for each hinge. Scale bar, 5 mm.

Figure 2 shows the modeling of the linkage mechanism of the origami-TEG and the design method of the hinge. Generally, in self-folding using heat-shrinkable film, it is obvious that the side to which the shrink layer is attached will be the valley side because the shrink layer folds up the non-shrinkable structure by the force of heat-induced shrinkage. Therefore, when the shrink layer is attached from only one side, both mountain and valley self-folding is impossible. As shown in Figure 2a, the gap in the structure of an origami-TEG is inaccessible after the TE element is mounted; thus, a heat-shrinkable film must be added externally on one side, which is usually difficult to achieve self-folding in a bellows fold. In this study, a pair of origami-TEG was modeled as a 6-bar link model consisting of six rigid bars and elastic hinges, and the driving force on one side of the mountain/valley folded up the hinge on the other side. Considering the balance of forces and moments at each rigid bar, it was possible to fold all six hinges to the designed angle with only three drives at hinges 1, 3, and 5. The theoretical driving force required for each hinge can be calculated by solving the equation in which the force is applied to each link and the moment balance of each joint because the active material is zero and the structure is in mechanical equilibrium after self-folding.

As shown in Figure 2b, assuming an elastic hinge, the moment *M* generated in the hinge is expressed as the product of the elastic modulus *k* of the hinge and folding angle *θ*. Thus, the driving force of the hinge can be controlled if the folding angle is controlled when the elastic modulus is constant. Therefore, we controlled the folding angle based on the attachment state of the heat-shrinkable film, as shown in Figure 2c. There were four types of shrink layers to be attached, namely I-, L-, T-, and cross-shaped. Parameter *L*_s_, which changes the attached area of the shrink layer, was defined for each type, with the driving force generated at the hinge changed by changing *L*_s_. The materials used to fabricate the test specimens and origami-TEG included a 33 µm thick polyimide copper film (Toray Advanced Materials Korea, Metaloyal^®^, Seoul, Republic of Korea) as the substrate, a 12 µm thick polyolefin film (Taiyo Electric Industry Co., HS-2520, Tokyo, Japan) as the active material, and 25 µm double-sided tape (NEION Film Coating Corp., MHM-SI25, Tokyo, Japan) as the adhesive layer joining the substrate and shrink layer. The crease portion of the substrate was cut using a UV laser machine (Osada Photonics International, OLMUV-355-5A-K) with a cut pattern that yielded a local reduction in the bending stiffness and electrical connection. The results of heating at 82 °C for 3 min in an oven (Yamato Scientific Co., DK300, Tokyo, Japan) with varying shrink layer parameters *L*_s_ are shown in Figure 2d. As shown in the photographs, the folding angle *θ* of both hinges increased as *L*_s_ increased. The trend in the relationship between *L*_s_ and *θ* was different for the specimen with the I-shaped shrink layer attached to the bottom substrate and the other three types of specimens attached to the top substrate. Therefore, an approximate curve was drawn from the experimental values of the I-shaped specimen and the other three types, with the shrink layer parameter *L*_s_ required for each hinge theoretically calculated.

## 3. Experiment and Result

**Figure 3:**
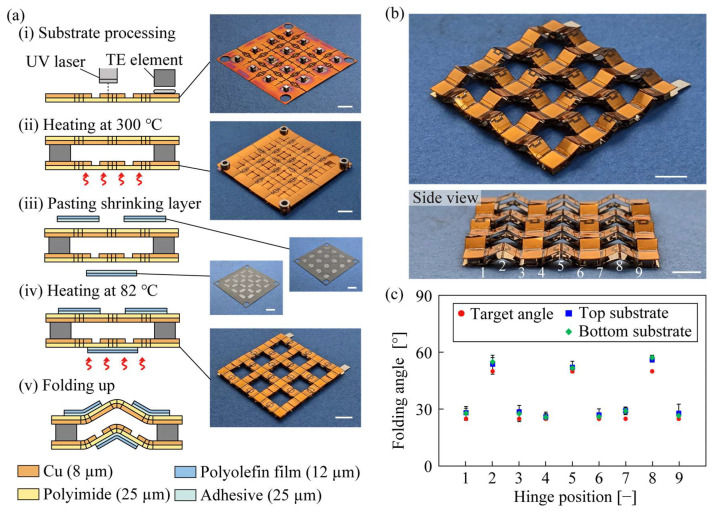
Fabrication process. (**a**) Detailed fabrication procedure of origami-TEG. (**b**) Photograph of origami-TEG after self-folding. (**c**) Accuracy of the self-folding angle of each hinge. The scale bar was 5 mm.

The fabrication process of the origami-TEG is illustrated in Figure 3. First, a polyimide/copper substrate was processed with a UV laser cutting machine to create a hinge cut and wiring patterns by scraping copper (Figure 3(a-i)). TE elements (manufactured by Toyoshima Seisakusho, p-type; Bi_0.3_Sb_1.7_Te_3_, n-type; Bi_2_Te_3_, 1.5 × 1.5 × 1 mm), which were pre-coated with special solder Cerasolza (Kuroda Techno Co., 168936, Yokohama, Japan) using an ultrasonic soldering machine (Kuroda Techno Co., USM-560) to improve their bite on the substrate, were mounted on a substrate coated with cream solder and heated to 300 °C on a hot plate (Figure 3(a-ii)). The shrink layer and double-sided tape were attached to a weak adhesive sheet and laser-cut at a power sufficient to cut only the shrink layer and double-sided tape, with the unwanted portions removed. The remaining pattern was transferred to the device (Figure 3(a-iii)). The fabricated device was again heated in an oven at 82 °C for 3 min (Figure 3(a-iv)). The polyolefin film shrunk by heat generated a driving force for folding, and the structure folded into the designed shape (Figure 3(a-v)). Figure 3b shows a photograph of the origami-TEG after self-folding. The size of the device before self-folding was 30 mm × 30 mm and after self-folding it was 27 mm × 27 mm. The overall weight of the device was 0.44 g and the weight per unit area was 0.06 g/cm^2^. No warpage or non-uniform folding of the device was observed, indicating that the device had self-folded almost as designed. Figure 3c shows the results of measuring the folding angles of the top and bottom substrates at each hinge position for detailed analysis. Hinges 1, 3, 4, and 6 were designed to fold up 25° from a flat position; the top substrate folded up with an average error of 3.2° and the bottom substrate with an average error of 3°. Considering that the angular dispersion of the hinge specimen was approximately 5.7°, it can be said that the proposed design method using the linkage mechanism fabricated the target origami structure with high accuracy. The bellows fold structure provided high stretchability to the top and bottom substrates, allowing the origami-TEG to follow any curved surface with non-zero Gaussian curvature without high stresses on the substrates.

**Figure 4:**
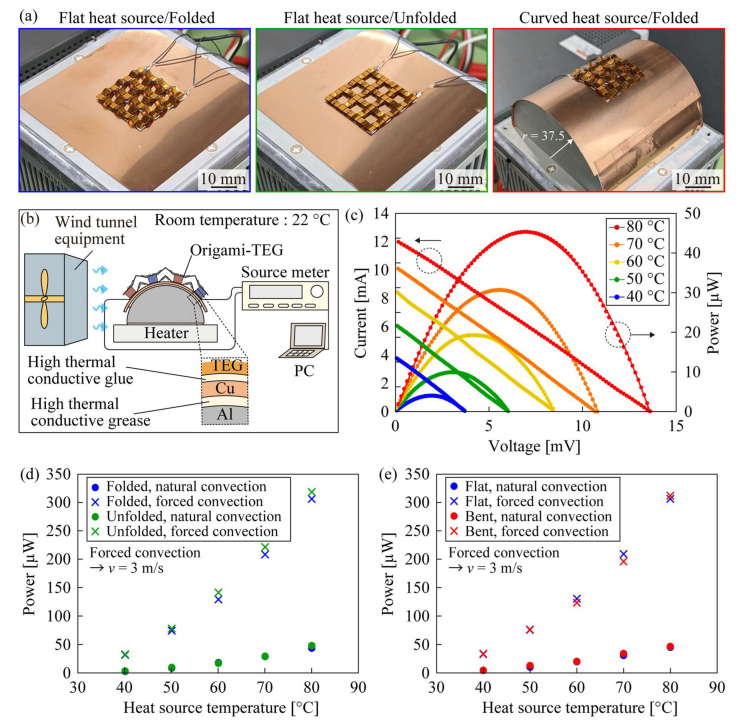
Performance evaluation of origami-TEG. (**a**) Photographs of three measured states of origami-TEG (**b**) Experimental setup during basic characteristics’ measurement. (**c**) Current-voltage and power-voltage characteristics of a folded origami-TEG when attached to a flat heat source. (**d**) Stability of output power against stretching deformation. (**e**) Stability of output power against bending deformation.

**Figure 5:**
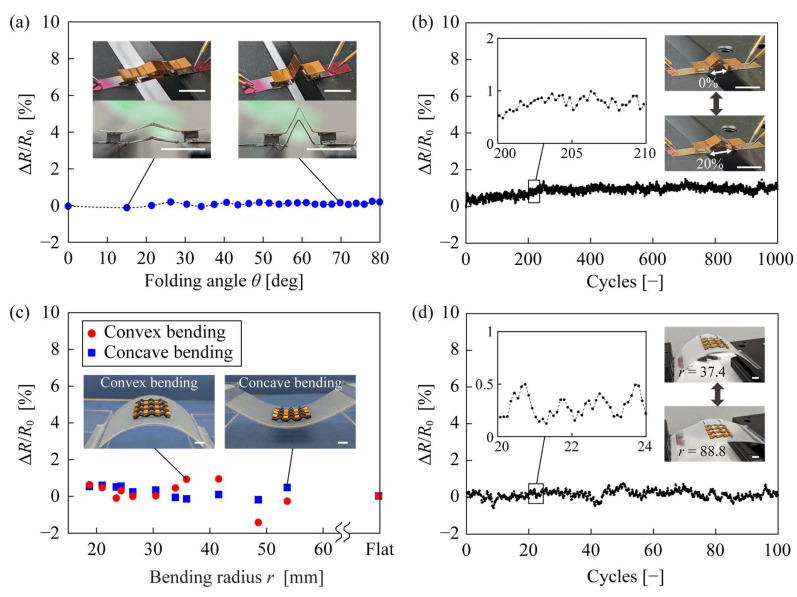
Evaluation of deformation resistance. (**a**) Resistance change when the folding angle is changed. (**b**) Cycle test with repeated stretching deformation at 20% strain. (**c**) Resistance change when bent in convex and concave directions. (**d**) Cycle test with repeated bending deformation. The scale bar was 5 mm.

To evaluate the performance of the fabricated origami-TEG, its basic characteristics were measured when attached to a high-temperature source, and the results are shown in Figure 4. As shown in Figure 5a, three cases were prepared: a self-folded origami-TEG attached to a flat heat source; an unfolded origami-TEG attached to a flat heat source; and a self-folded origami-TEG attached to a curved heat source. The experimental setup for performance evaluation is shown in Figure 4b. The flexibility of the origami-TEG allows it to be attached to surfaces of various shapes and materials and, in this study, a copper film with high thermal conductivity was chosen as the attachment surface to enable an accurate measurement of the characteristics. The origami-TEG was fixed on a copper film with highly thermally conductive glue (Cemedine Co., SX1008, Tokyo, Japan) and attached to a heat source coated with highly thermally conductive grease (Shin-Etsu Silicone, G-747, Tokyo, Japan). The temperature of the heat source varied by 10 °C between 40–80 °C using a Peltier temperature controller (VICS, VTH1.8K-70S). The *I*–*V* characteristics were measured using a source meter (Keithley Instruments, 2614 B, Cleveland, OH, USA) under both natural and forced convection at a wind speed of 3 m/s using wind tunnel equipment (Kanomax, S0522195, Osaka, Japan). A semi-cylindrical aluminum block with a radius of 37.5 mm was prepared as a curved heat source. The basic characteristics of the self-folded origami-TEG under natural convection are shown in Figure 4c. The *I*–*V* characteristics were linear at each temperature, while the output power increased as the heat source temperature increased, with a maximum power output of 44.7 µW at an 80 °C heat source temperature.

Figure 4d compares the maximum output power of the origami-TEG in folded and unfolded states when attached to a flat heat source. The average relative difference in the maximum output power between the two states was 8.5% under natural convection and 5.3% under forced convection. No significant performance advantage was observed when the device was folded, indicating that the origami structure can provide stretchability to the TEG without affecting its performance. Figure 4e compares a self-folded origami-TEG attached to flat and curved heat sources. The average relative difference in the maximum output between the two states was 10.9% for natural convection and 3.2% for forced convection, confirming that the effect on performance when attached to a curved heat source is negligible. These results demonstrate that the origami-TEG can be used as flexible TEGs under both stretching and bending deformations.

Finally, we confirmed the stability of the internal resistance when the origami-TEG was deformed; the results are shown in Figure 5. Figure 5a shows the change rate of the internal resistance (Δ*R*/*R*_0_) of one unit of origami-TEG when the folding angle of the substrate was changed, with a maximum value of 0.2%, indicating high stability under stretching deformation. The stretch ratio from the folding angle of 0° to 80° is 83%, characterizing the high stretchability of origami-TEG, given that most conventional flexible TEGs have a maximum stretch ratio of less than 50% strain [6,7,8]. The change in resistance was stable even after 1000 cycles of deformation at 20% strain stretching, as shown in Figure 5b. Figure 5c shows the results of measuring Δ*R*/*R*_0_ when the entire device was bent in the convex and concave directions at bending radius *r*. Δ*R*/*R*_0_ shows a low value of 0.6% for both convex and concave bending, even when bent to *r* = 18.6 mm. As shown in Figure 5d, the internal resistance remained almost unchanged after 100 bending cycles, which deformed *r* from 37.4 mm to 88.8 mm. The origami-TEG was deformed only once when it was manually attached to the curved high-temperature source, or several times if reattachment is considered. Therefore, the two cycle-test results in Figure 5 indicate that the device is sufficiently robust for practical use. These results demonstrate that the origami-TEG has high internal resistance stability for both stretching and bending deformations.

## 4. Conclusions

In this study, a flexible TEG with an origami structure (origami-TEG) was fabricated by self-folding. By applying the modeled device structure to the design theory of self-folding using a linkage mechanism, it is possible to fold the device into the target bellows-folded structure even when the attachment of the active material is restricted. The output power and change rate of the internal resistance of the TEG during deformation was measured, and it was confirmed that the TEG had high-performance stability and mechanical reliability for both stretching and bending deformations. In conclusion, the proposed self-folding method achieved the fabrication of an origami-TEG that enables both flexibility and the usage of high-performance rigid materials. This study clearly shows the fabrication method and usefulness of TEG with an origami structure, possibly paving the foundation to realize high-performance flexible TEGs using high-performance bulk TE elements, such as BiTe-based materials and substrate materials with high thermal conductivity and no stretchability, which are difficult to achieve with conventional approaches.
